# Successful management of severe acute respiratory distress syndrome caused by sodium polystyrene sulfonate aspiration: A case report

**DOI:** 10.1097/MD.0000000000016574

**Published:** 2019-07-26

**Authors:** Cheng-Yu Ko, Ping-Yen Liu, Po-Wei Chen

**Affiliations:** a Division of Cardiology, Department of Internal Medicine, National Cheng Kung University Hospital, College of Medicine; b Institute of Clinical Medicine, College of Medicine, National Cheng Kung University, Tainan, Taiwan.

**Keywords:** acute respiratory distress syndrome, extracorporeal membrane oxygenation, Kayexalate, sodium polystyrene sulfonate

## Abstract

**Rationale:**

Sodium polystyrene sulfonate is commonly administered to treat hyperkalemia. Severe pneumonia due to aspiration of this drug is rare and no survival case has thus far been reported.

**Patient concerns:**

A 45-year-old man was hospitalized for acute decompensated heart failure and acute kidney injury with hyperkalemia. He aspirated sodium polystyrene sulfonate while consuming the drug. Severe acute respiratory distress syndrome (ARDS) developed rapidly, and he was transferred to the intensive care unit (ICU).

**Diagnoses:**

Chest radiography results after aspiration showed new consolidation in the left upper lung. He underwent emergency bronchoscopy, which revealed a considerable amount of yellow mud-like material in the trachea and bronchi. Chest radiography results after the bronchoscopic removal of the foreign material revealed rapid resolution of the left upper lung consolidation.

**Interventions:**

In the ICU, mechanical ventilation with low tidal volume and high positive end-expiratory pressure was administered and extracorporeal membrane oxygenation (ECMO) was set up for treating severe ARDS. We arranged an emergency bronchoscopy for diagnosis and removal of polystyrene sulfonate.

**Outcomes:**

ECMO was discontinued after 10 days and the patient was discharged after approximately 2 weeks.

**Lessons:**

Aspiration of sodium polystyrene sulfonate is not common but can be lethal. Clinicians should be cautious and appropriately inform patients of the aspiration risk while administering this drug. Mechanical ventilation and bronchoscopy were effective treatments for severe ARDS caused by aspiration of this drug.

## 1 Introduction

Sodium polystyrene sulfonate is used for the treatment of hyperkalemia and is a cation-exchange resin administered as a suspension either orally or through retention enema.^[1]^ Oi^[2]^ first described a case of neonatal death associated with sodium polystyrene sulfonate (Kayexalate) aspiration in 1978. Since then, sporadic cases of pneumonitis or bronchitis associated with aspiration of this substance have been reported incidentally in autopsy findings and pathological specimens. Immediate morbidity has not been attributed to polystyrene sulfonate aspiration in most of the reported patients^[3–5]^; Duggal^[6]^ reported the first case in the English language literature of severe acute respiratory distress syndrome (ARDS) associated with aspiration of sodium polystyrene sulfonate. We report the case of a patient who survived severe ARDS caused by sodium polystyrene sulfonate aspiration.

### 1.1 Patient information and clinical findings

A 45-year-old man was hospitalized for acute decompensated heart failure. He had a history of heart failure with reduced ejection fraction (left ventricular ejection fraction = 29% on echocardiography 1 month before hospitalization) and mitral valve regurgitation status post valve annuloplasty. He presented with progressive dyspnea, decreased urine output, and edema of the legs since 1 week before admission.

At admission, his hemodynamics was stable; however, physical examination revealed bilateral lung crackles on auscultation, cold limbs, and edema on both legs. Laboratory analysis results revealed increased levels of N-terminal pro b-type natriuretic peptide without anemia or leukocytosis. The chest radiograph revealed cardiomegaly, pulmonary edema, and right pleural effusion. Inotropic agents and diuretics were administered for heart failure.

**Figure 1:**
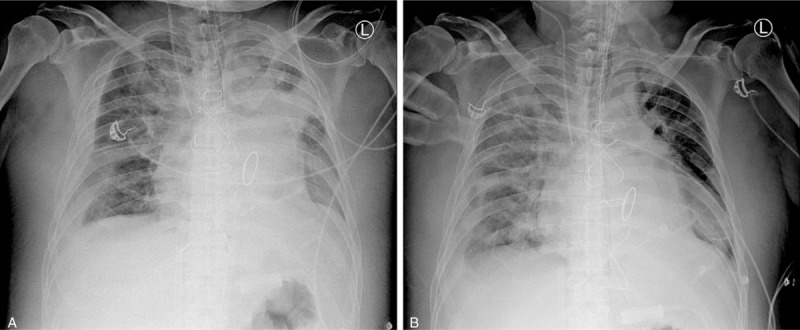
A, Chest radiograph after sodium polystyrene sulfonate aspiration exhibited a new consolidation in the left upper lung. B, After bronchoscopic removal of the foreign material, rapid resolution of left upper lung consolidation was observed 4 hours after the aspiration episode.

During hospitalization, his renal function deteriorated and follow-up laboratory analysis revealed hyperkalemia; consequently, sodium polystyrene sulfonate was administered. He consumed the powder of polystyrene sulfonate without diluting it with water and he choked on the powder. The patient had hypoxemia and respiratory distress, intubation was arranged, and he was transferred to the intensive care unit (ICU). The chest radiograph revealed a new dense consolidation in the left upper lung (Fig. 1A).

### 1.2 Interventions

**Figure 2:**
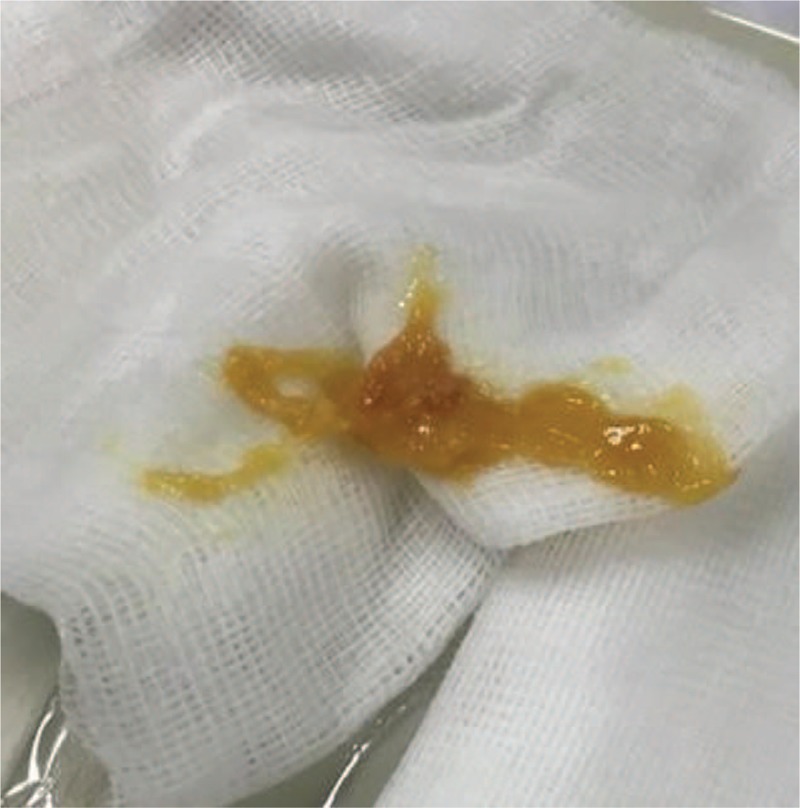
Yellow mud-like material that appeared similar to the powder of sodium polystyrene sulfonate was removed using emergency bronchoscopy.

In the ICU, he soon developed ARDS and was treated with mechanical ventilation with a low tidal volume and high positive end-expiratory pressure. The patient was sedated and exhibited paralysis. Because the patient exhibited severe ARDS and highly rapid exacerbation, extracorporeal membrane oxygenation (ECMO) was arranged for him. Emergency bronchoscopy revealed a considerable amount of yellow mud-like material (Fig. 2) in the trachea and left main bronchus; the left segmental bronchi were also obstructed by the foreign material. The foreign material was removed using bronchoscopic suction, and the chest radiograph after suction showed rapid resolution of the left upper lung consolidation (Fig. 1B).

### 1.3 Follow-up and outcomes

With ECMO support and the use of inotropic agents and antibiotics, the patient's condition improved gradually. ECMO was discontinued after 10 days and the sedative agents were discontinued because the patient exhibited considerably improved oxygenation.

## 2 Discussion

This case of severe ARDS associated with aspiration of sodium polystyrene sulfonate is unique in 3 respects. First, our patient survived owing to emergent ECMO support, antibiotic use, and bronchoscopic removal of the aspirated material. In contrast, almost all previously reported patients did not survive aspiration episodes with severe hypoxemia and fulminant clinical course. ECMO or emergent bronchoscopy was not offered in previously reported patients.^[3–6]^ Acute respiratory failure associated with aspiration of sodium polystyrene sulfonate is unique and rapidly progressive. Emergent ECMO support and multidisciplinary care teams on intensive care played critical roles to stabilize hemodynamic condition in this kind of situation.

Second, our patient was a middle-aged man with independent daily activities and not a debilitated elderly patient at a relatively high risk of aspiration or choking, as has been typical in previously reported cases.^[3,4,6]^ Third, in this study, the hypoxemia episodes immediately after polystyrene sulfonate administration and the considerable improvement in chest radiography results before and after bronchoscopic suction confirmed the diagnosis, whereas in previous studies the relationship between pneumonia and aspiration of polystyrene sulfonate was confirmed during autopsy or after obtaining pathology results.^[3,4,6]^

Previous report documented that polystyrene sulfonate aspiration is accompanied by florid polymorphonuclear pulmonary infiltrates, suggesting that the resin causes lung injury.^[7]^ Clinicians should be cautious when the drug is prescribed, irrespective of whether a patient has a high or low risk of aspiration, because refractory hypoxemia and even ARDS might occur. An unremarkable medical behavior could result in a catastrophe.

For the management of ARDS associated with aspiration of polystyrene sulfonate, the mechanical ventilation settings need not necessarily be different from the general principles recommended for ARDS, namely low tidal volume and open lung ventilation. Bronchoscopic removal of the foreign material was a critical step that was also described in a previous case report.^[6]^ A combination of empirical antibiotic treatment, bronchoscopic suction, and mechanical support, namely ventilator use and ECMO, was critical for the survival of the patient.

In conclusion, we highlight that sodium polystyrene sulfonate aspiration can occur in normally active patients and even cause medical catastrophes such as ARDS. Clinicians should exercise caution inform patients of the consequences of aspiration while administering this drug. Both mechanical support and bronchoscopy are practical methods for treating this condition.

## Author contributions

**Conceptualization:** Po-Wei Chen.

**Data curation:** Po-Wei Chen.

**Formal analysis:** Po-Wei Chen.

**Investigation:** Cheng-Yu Ko, Ping-Yen Liu, Po-Wei Chen.

**Project administration:** Po-Wei Chen.

**Writing – original draft:** Cheng-Yu Ko, Po-Wei Chen.

**Writing – review and editing:** Ping-Yen Liu, Po-Wei Chen.

Po-Wei Chen orcid: 0000-0003-2300-0698.
